# TGF-beta1 on osteoimmunology and the bone component cells

**DOI:** 10.1186/2045-3701-3-4

**Published:** 2013-01-15

**Authors:** Shimpei Kasagi, Wanjun Chen

**Affiliations:** 1 Mucosal Immunology Section, NIDCR, NIH, Bethesda, MD, USA

## Abstract

TGF-β1 is an immunoregulatory cytokine that regulates immune cell proliferation, survival, differentiation, and migration. Compelling evidence has demonstrated a strong association between the immune and skeletal systems (so called Osteoimmunology), such as the critical role of TGF-β1 in the development and maintenance of the skeletal tissue. This review provides an overview of the mechanisms in which TGF-β1 interacts with bone component cells, such as osteoblasts, osteoclasts, chondrocytes, mesenchymal stem cells, and hematopoietic stem cells, in concert with other cytokines and hormones.

## Introduction

Bone is a rigid organ that constitutes part of the endoskeleton of vertebrates. It serves multiple functions; providing mechanical support for joints and tendons, protecting soft tissue or various organs from mechanical stress or trauma, storing minerals, generating hematopoietic cells, and producing hormones. These many functions are regulated by several soluble factors. Interestingly, accumulated evidence indicates that transforming growth factor beta 1 (TGF-β1) plays a critical role in bone formation, mineral storage, and hematopoietic cell generation. In addition, recent progress in the study of the cross-talk between the skeletal system and the immune system (termed osteoimmunology) has revealed shared components and mechanisms between the two systems [1]. This review highlights recent findings focusing on the role of TGF-β1 in bone metabolism and osteoimmunology.

**Figure 1:**
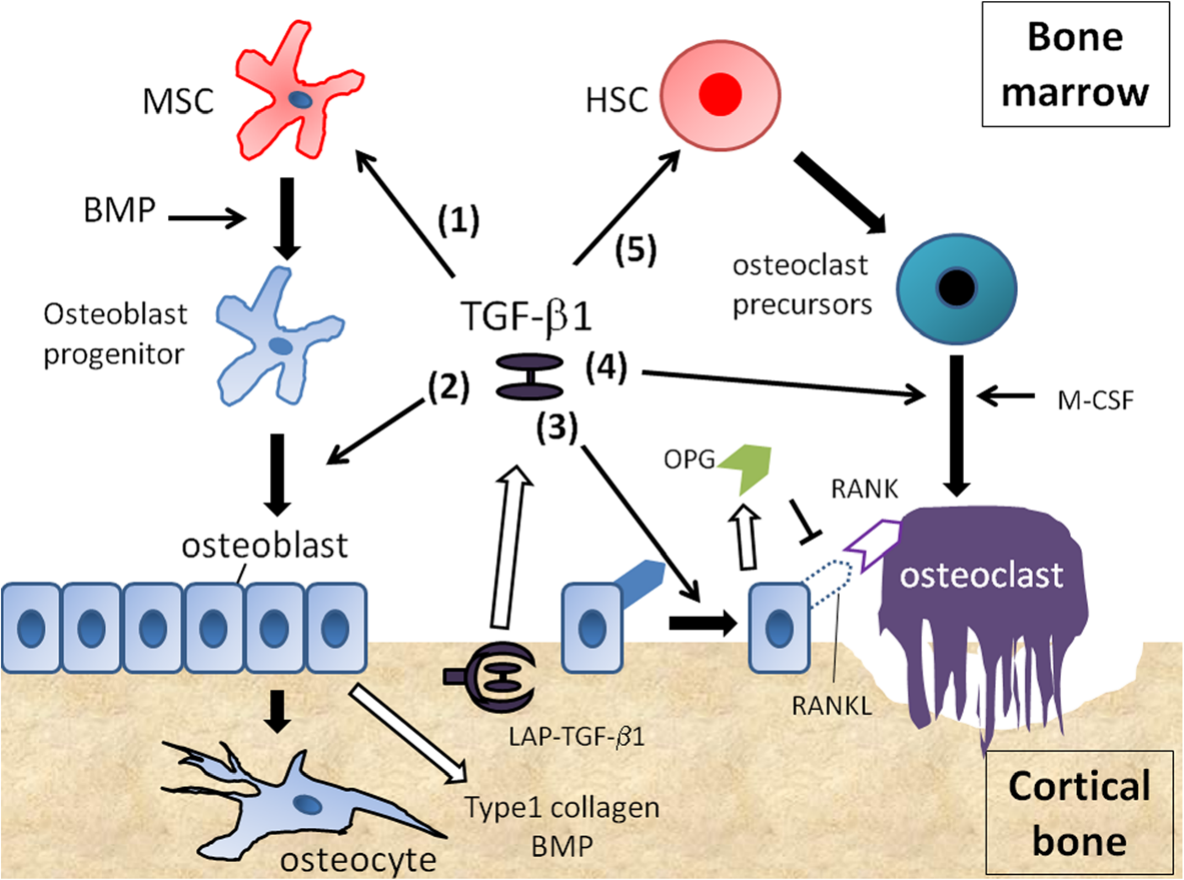
**Five major role of TGF**-**β1 in osteoimmunity are shown. **(1) TGF-β1 stimulates the proliferation of MSCs, and promotes their differentiation into chondrocytes. (2) TGF-β1 promotes osteoblast progenitor’s differentiation into osteoblast. (3) High concentration of TGF-β1 enhances osteoblast proliferation, and downregulates the expression of RANKL of osteoblast. (4) Low concentration of TGF-β1 promoted osteoclast maturation. (5) TGF-β keeps hematopoietic stem cells (HSC) in hibernation state.

### Function of TGF-β1 in the immune system

TGF-β1 is a one of the most potent regulatory cytokines with diverse effects on hematopoietic cells. In the immune system, TGF-β1 induces and maintains immune tolerance by regulation of lymphocyte proliferation, differentiation, and survival. Disruption of TGF-β1 results in a dysregulated immune system [2], leading to inappropriate immune cell activation, inflammation, cancer (Leukemia and malignant lymphoma etc) and autoimmune diseases [3]. In fact, TGF-β1-deficient mice (TGF-β1ko) die within 2-3 weeks after birth due to lymphocyte and monocyte infiltration of multiple vital organs [3,4]. CD4^+^Foxp3^+^ T cells that predominantly produce anti-inflammatory cytokines (TGF-β, IL-10), but not inflammatory cytokines (IFN-γ, TNF-α, and IL-17) are known as regulatory T cells (Tregs). They inhibit the action of other effector T cells through soluble factors (TGF-β or IL-10) or cell-to-cell contact [5]. It is well known that TGF-β suppresses inflammatory cytokine production by effector T cells, and additionally, it drives the differentiation of naïve T cells to CD4^+^CD25^+^Foxp3^+^ Tregs [6]. Moreover, TGF-β controls the initiation and resolution of inflammatory responses through the regulation of chemotaxis, activation, and survival of lymphocytes [2], natural killer cells [7], dendritic cells [8], macrophages [9], mast cells [10], and granulocytes [11]. These findings indicate that TGF-β plays critical roles in the regulation of immune responses.

### Bone component cells in bone remodeling

Bone remodeling is a lifelong process where mature bone tissue is removed from the skeleton (a process called bone resorption) and new bone tissue is generated (a process called bone formation). An imbalance between bone resorption and bone formation results in many metabolic bone diseases, such as osteoporosis and Camurati-Engelmann disease [12,13]. The cells responsible for bone formation are known as osteoblasts which secrete new bone, and those responsible for bone resorption are known as osteoclasts, which break bone down. Osteoblasts arise from mesenchymal stem cells (MSCs) located in bone marrow, and Osteoclasts are generated from hematopoietic stem cells (HSCs). TGF-β1 controls both osteoblast and osteoclast differentiation, and therefore balances bone formation and resorption [14] (Figure 1).

The TGF-β family contains three closely related mammalian isoforms—TGF-β1, -β2, and -β3, yet TGF-β1 constitutes the largest sources of TGF-β in bone [15]. In mice, TGF-β1 protein is detected in bone marrow cells, chondrocytes, and the cartilaginous matrix [16]. As discussed in the next chapter, TGF-β1 regulates bone formation through enhancement of osteoblast proliferation [17], differentiation[18], and chemotactic attraction[19], and also extracellular matrix and proteoglycan synthesis of chondrocyte precursor cells [20]. We will discuss how TGF-β1 maintains bone homeostasis through regulating bone-forming cells, individually.

### TGF-β1 and osteoblasts

Osteoblasts are mononucleated cells that are responsible for bone formation. They arise from osteoblastic precursors located in the deeper layer of periosteum and the bone marrow, and produce a matrix of osteoid, which is composed mainly of type I collagen [21]. TGF-β1 has a variety of widely recognized roles in bone formation. For example, TGF-β1 enhances osteoblast proliferation [17], blocks apoptosis of osteoblasts [18], and also recruits osteoblastic precursors or matrix-producing osteoblasts to the site through chemotactic attraction [19]. In addition, TGF-β1 enhances the production of extracellular bone matrix protein by osteoblasts in the early stages of osteoblast differentiation [22]. On the other hand, TGF-β1 inhibits the later phase of osteoblast proliferation and mineralization [23]. It has been previously reported that both TGF-β receptor I and receptor II expression in murine, rat, and human osteoblasts was decreased during osteoblast differentiation, which may imply that osteoblasts are less sensitive to TGF-β1 in the late phase of their differentiation [24]. The later stages are positively regulated by bone matrix proteins (BMP), which are members of the TGF-β superfamily [25]. Therefore, TGF-β1 cooperates with BMP to regulate the differentiation of osteoblasts.

Runt-related transcription factor 2 (Runx2), also known as core-binding factor subunitalpha-1 (Cbfa1), is a DNA-binding transcription factor important in specifying osteogenic lineage [26]. Runx2 is the master transcription factor in bone formation, and several reports indicated that it is regulated by TGF-β1 and BMP-2. In the initial phase of osteoblastic differentiation (differentiation of MSCs to osteoblast progenitor cells), Runx2 inhibited differentiation of MSCs to types of cells other than osteoblasts, which required coordinated action between Runx2 and BMP2-induced Smad5 [27]. In the second phase of their differentiation (from osteoblast progenitor cells to osteoblasts), TGF-β1 induced the expression of Runx2, which cross-talks with beta-catenin signaling to promote differentiation [28]. However, in the final differentiation stages of osteoblasts (mature osterblasts), TGF-β1 opposes BMP-2 actions [27]. Smad3, activated by TGF-β1, physically interacts with Runx2 at Runx2-responsive elements, and suppresses the expression of Runx2. Runx2 prevents the differentiation of mature osteoblasts into osteocytes, and maintains them in a resting state. Thus, TGF-β1 may promote osteocyte development by downregulating Runx2 in mature osteoblasts [18]. These findings indicate that TGF-β1 finely tunes osteoblast differentiation.

### TGF-β1 and osteoclasts

The role of TGF-β1 in osteoclastogenesis and bone resorption is very complex and controversial. It is clear that TGF-β1 mediates osteoclasts functions, such as their maturation [29], apoptosis [30], and the recruitment of osteoclast precursors from spleen or bone marrow [31]. Several reports indicated that TGF-β1 had a biphasic effect on the osteoclast maturation. TGF-β1 induces osteoclastogenesis of hematopoietic precursor and other osteoclastic precursors when it is added into the culture with receptor activator of nuclear factor kappa B ligand (RANKL) and macrophage colony stimulating factor (M-CSF) [32]. TGF-β1 triggers the expression of nuclear kappa B (NF-κB) and receptor activator of nuclear factor kappa B (RANK) in osteoclast precursors, and RANKL-RANK interaction is important for prolonged survival and augmented differentiation of osteoclast precursors into osteoclasts [32]. On the other hand, when osteoclast precursors were cultured with osteoblasts, it seemed that osteoclast activation was attenuated especially when they were stimulated with high concentrations of TGF-β1 (1-10 ng/ml), while low concentrations of TGF-β1(1-10 pg/ml) promoted osteoclast maturation [33]. High concentrations of TGF-β1 up-regulated the expression and secretion of osteoproteogerin (OPG) and down regulated that of RANKL by osteoblasts [34]. OPG is a decoy receptor for RANKL, and inhibits the RANKL-RANK interaction by binding to RANKL on osteoblasts [35]. As a result, the differentiation of osteoclast precursors into mature osteoclasts mediated by RANKL-RANK is inhibited by OPG released from osteoblasts.

### RANKL-RANK-OPG axis and osteoimmunology

The RANKL-RANK-OPG axis is an important signaling system functioning both in bone and immune cell communication. RANKL expressed on T cells or soluble forms of RANKL are able to induce matured osteoclasts. RANK is also expressed on dendritic cells, and RANKL activates dendritic cells by binding to RANK. RANKL/RANK signaling inhibited apoptosis of dendritic cells, resulting in an increase in dendritic cell-mediated T cell proliferation in a mixed leukocyte reaction [36]. OPG is expressed on monocytes, T cells and B cells [37]. Interestingly, cytokines stimulating the osteoclastogenesis, such as IL-1β, IL-6, IL-17, and TNF-α increased the expression of RANKL with decrease of OPG expression in osteoblasts [37]. These findings suggest that RANKL-RANK-OPG axis may regulates immune responses. More recently, RANKL-RANK-OPG axis has been reported to be involved in the pathogenesis of degenerative bone diseases, such as rheumatoid arthritis and psoriatic arthritis, post menopausal osteoporosis, and bone metastasis [37,38]. Novel strategic treatments are emerging that are based on an understanding of the functional status of the OPG/RANK/RANKL triad.

### TGF-β1 and mesenchymal stem cells

MSCs are muitipotent progenitor cells that have the ability to differentiate into mesenchymal lineages, including osteoblasts (bone), chondrocytes (cartilage) and adipocytes [39]. The differentiation, and function of MSCs can be regulated by TGF-β1. For example, TGF-β1 stimulates the proliferation of MSCs and promotes their differentiation into chondrocytes [40]. TGF-β1 promotes chondrogenesis as well as the early phase of osteogenesis, however in the absence of BMPs, TGF-β1 is unable to promote differentiation of MSCs into osteoblasts as described previously [27].

MSCs have potent immunosuppressive effects through cell-to-cell contact and by secreting soluble factors. They have a clear potential for clinical application for the repair of damaged tissue [41] and in the therapy of autoimmune diseases and chronic inflammation [42]. Soluble factors secreted by MSCs include indoleamine 2,3-dioxygenase (IDO), prostaglandin E2 (PGE2), and TGF-β which all plays a major role in MSC-mediated suppression of T cell proliferation induced by mitogens or alloantigens [43,44]. IDO metabolizes tryptophan to kynurenine, which causes depletion of local tryptophan and reduction of lymphocyte proliferation [45,46]. PGE2 is a powerful mediator that inhibits T cell mitogenesis and IL-2 production [47]. Park et al. recently reported that TGF-β1-transduced MSCs, but not control MSCs, suppressed the development of type II collagen-induced arthritis (CIA). *In vivo* treatment with TGF-β-transduced MSCs reduced bone erosion and cartilage destruction by suppressing type II collagen-specific T cell proliferation, by down-regulating proinflammatory cytokine production (IL-6, TNF-α, and IL-17), and by increasing the number of type II collagen-specific CD4^+^Foxp3^+^ Tregs in the spleen [48]. These findings suggested that TGF-β produced by MSCs *in vivo* could play a key role in peripheral tolerance and bone repair.

### TGF-β And hematopoietic stem cells in bone marrow

Hematopoiesis is the formation of blood cellular components (red blood cells, white blood cells, and platelets), and critical to maintain their number in the peripheral circulation. Hematopoietic stem cells (HSCs) reside and self-renew in the bone marrow (BM) niche, and have the unique ability to give rise to all of the different mature blood cell types. The term ‘BM niche’ was introduced in the 1980s to define the spatial and temporal structure harboring stem cells [49]. Many different cell types have been characterized as contributing to the formation of HSC BM niches, such as osteoblasts, endothelial cells, MSCs, and reticular cells [50]. In the BM niche, most of the HSCs are kept in hibernation or undifferentiated states and few cells are recruited into the cell cycle at long intervals, on average every 1 to 2 months [51]. Tight regulation of HSCs’ proliferation and differentiation is vital to retain the high quality of HSCs. This process is also critical to avoid autoimmune diseases or hematologic malignancy triggered by abnormal HSCs released from BM to periphery. HSCs outside the niche do not self-renew and commence the process of differentiation to produce mature blood cells [51], indicating that the presence of BM niche is critical for HSCs homeostasis.

Recently, TGF-β has been reported to contribute to the formation of HSC niches. Yamazaki et al. reported that TGF-β produced by glial cells (one of the components of BM niche) was critical for the maintenance of BM niche [52]. They demonstrated that TGF-β type II receptor-deficient HSCs showed impaired long-term self-renewal activity. They also demonstrated that latent TGF-β, produced by a variety of BM cells, was broadly distributed within BM, while, active TGF-β was exclusively detectable in glial cells that lay in parallel with blood vessels. Since two distinct areas (perivascular area and endosteal area) have been characterized as HSCs niches [50], their finding suggested that active TGF-β released by glial cells has a critical role in the perivascular BM niche. MSCs and osteoblasts, which are the components of the endosteal niche, recruit HSCs and maintain their survival by producing HSCs promoting factors [50]. However, it remains unknown if TGF-β produced by MSCs is critical for the maintenance of BM niche. Further investigation is required to establish this new concept.

### TGF-β and T cells in osteogenesis

The role of TGF-β signaling in T cells and the effect of this upon osteogenesis was previously investigated by Gao et al [53]. They reported that disruption of T cell specific TGF-β signaling led to bone loss. T cells purified from CD4 dominant negative TGF-β RII mice produced more TNF-α and RANKL than wild type mice. These factors, in concert, stimulated osteoclasts formation and activity, which resulted in impaired skeletal maturation and bone loss in estrogen repleted mice (osteoporosis model mice). *In vivo* treatment with a TGF-β1 expression vector restored bone loss through deactivating effector T cell function, suggesting that TGF-β1 signaling in T cells could modify bone metabolism. In addition to the effects of TGF-β1, TGF-β2 also influences T cells effctor function which has implications for osteogenesis. TGF-β2 inhibits T cell activation through TGF-βR2, suggesting that the absence of TGF-β2-mediated signaling may be a candidate of dysregulated production of TNF-α and RANKL in CD4 dominant negative TGF-β RII mice. Notably, it has been reported that TGF-β2 stimulates synthesis of TGF-β1 in chondrocytes and osteoblasts within newly generated bone and cartilage, indicating a positive autoregulation of TGF-β [54]. Together, these results suggest that TGF-β2 signaling play a regulatory role in T cell activation and bone metabolism.

TNF-α has been previously reported to promote osteoblast apoptosis [55] and osteoclasts genesis from bone marrow macrophages [56]. TGF-β1 attenuates TNF-α-induced osteoblasts apoptosis and bone resorption [57]. Since TNF-α plays a pathological role in the development of rheumatoid arthritis (RA), patients with severe RA are treated with anti-TNF-α therapy [58]. Interestingly, it has been reported that anti-TNF-α therapy enhanced FoxP3^+^ regulatory T cells in patients with RA via induction of TGF-β [59]. Thus, these data suggest that TGF-β protects bone from TNF-α induced bone damage by suppressing effecter T cell function and/or promoting induction of regulatory T cells. Besides TNF-α IL-6, IL-17, and IL-23 can induce osteoclast differentiation resulting in bone resorption [60]. For instance, it has been reported that IL-17 promotes osteoclastgenesis via RANKL induction on osteoblasts [61]. Low levels of TGF-β synergize with IL-6 to induce Th17 differentiation, whereas high levels of TGF-β result in development of Foxp3^+^ regulatory T cells [62]. Furthermore, IL-17 and TNF-α have been shown to induce IL-6 and IL-23 expression in synovial fibroblast [63]. These findings could suggest that TGF-β regulates osteoclast genesis by controlling Th17 differentiation.

RA is a disease mediated by both Th1 and Th17 cells, as both cell types accumulate in the joints of CIA mice [64]. In general, TGF-β inhibits Th1 cells differentiation, however, recent findings indicated that RA is not a Th1-mediated disease. For example, IFN-γ (Th1 related cytokine) was hardly detected in the synovial fluids of RA patients [65]. Moreover, IFN-γ has been shown to inhibit osteoclasts genesis in human [66]. Thus, the role of Th1 and Th17 cells in RA is still an open question.

### TGF-β1 and regulatory T cells in osteogenesis

Tregs induce and maintain immune tolerance, however several groups recently reported that regulatory T cells also promote bone formation. We have recently shown that CD4^+^ Foxp3^+^ Tregs improved MSCs-mediated bone formation [67] by suppressing the number of infiltrating neutrophils in recipients, and the levels of IFN-γ, IL-6, and TNF-α in the MSCs implants. However, IL-4 and IL-10 levels were not affected. These findings suggest that Tregs-mediated anti-inflammatory milieu could promote favorable conditions for bone regeneration. Zaiss et al. reported that Foxp3-Tg mice that overexpress Foxp3 had higher bone mass and were protected from ovariectomy-induced bone loss [68]. The increase in bone mass resulted from impaired osteoclasts differentiation and bone resorption *in vivo*, not from up-regulation of bone formation. Surprisingly, adoptive transfer of regulatory T cells into RAG-1^-/-^ mice (mice who have no mature T and B cells in lymphoid organs) increased the bone mass, indicating that regulatory T cells could directly affect bone homeostasis without other T cell lineages engagement. Collectively, these findings provide evidence that regulatory T cells promoted bone formation.

### TGF-β1 and Hormones

TGF-β1 interacts with soluble factors like hormones, such as estrogen, gluco-corticoids, parathyroid hormone (PTH), and vitamin D. Estrogen stimulates TGF-β1 production in osteoblasts, and promotes osteoblasts proliferation and differentiation. Moreover, estrogen prevents bone loss by promoting osteoclasts apoptosis through a TGF-β dependent mechanism [69]. Estrogen receptor α (ER- α) was identified as a co-repressor for Smad activity, and Smad3 was identified as an enhancer of ER-α mediated transcriptional activity, indicating that Estrogen and TGF-β1 coordinate actions during bone formation [70].

Gluco-corticoids are known to promote apoptosis of osteoblasts and also inhibit their proliferation and differentiation while promoting the differentiation of osteoclasts [71]. Gluco-corticoids upregulate TGF-β1 expression in osteoblasts, however, unlike estrogen, Gluco-corticoids synergise with TGF-β enhanced osteoclast formation by stimulating the priming of osteoclast progenitors for differentiation into osteoclasts [72]. PTH, a polypeptide secreted by parathyroid glands, enhances bone formation by increasing TGF-β mediated type I collagen production in osteoblasts. However, PTH concomitantly promotes bone resorption by binding to PTH receptor on osteoblasts and stimulating osteoblasts to increase their expression of RANKL but inhibits their expression of OPG [73]. Thus, PTH functions as a double-edged sword for bone metabolism.

Vitamin D is an important regulator of calcium homeostasis in bone. It stimulates bone formation by promoting osteoblast differentiation and extracellular matrix mineralization. Moreover, it inhibits PTH-mediated bone resorption [74]. Vitamin D receptor (VDR) is expressed on osteoblast precursors, and vitamin D signals via VDR result in up-regulation of a bone formation gene, osteocalcin [75]. Osteocalcin released by osteoblast precursors induces maturation of osteoblasts [76]. The effect of TGF-β1 on vitamin D functioning seems to diverge at the Smad3/MAPK level. Smad3 has been shown to act as a coactivator of vitamin D signaling to activate osteocalcin expression in a study using fibroblasts [77]. Interestingly, Smad3 binding elements and vitamin D responsive elements are closely located in the osteocalcin promoter [77]. On the other hand, TGF-β1 mediated AP-1/MAPK signaling pathways seem to antagonize osteocalcin synthesis in an osteosarcoma cell line [78]. In conflicting reports using mouse osteoblasts, TGF-β1 and vitamin D have been shown to both synergize and antagonize each other in bone formation [79,80]. Thus, it appears that signals transmitted through Smad3 and MAPK pathways evoke opposite effects on vitamin D functioning.

## Competing interests

The authors declare that they have no competing interest.

## Authors’ contributions

SK drafted and WJC edited the manuscript. Both authors have read and approved the final manuscript.
